# Guidelines for the management of osteoporosis and fragility fractures

**DOI:** 10.1007/s11739-018-1874-2

**Published:** 2018-06-13

**Authors:** Ranuccio Nuti, Maria Luisa Brandi, Giovanni Checchia, Ombretta Di Munno, Ligia Dominguez, Paolo Falaschi, Carmelo Erio Fiore, Giovanni Iolascon, Stefania Maggi, Raffaella Michieli, Silvia Migliaccio, Salvatore Minisola, Maurizio Rossini, Giuseppe Sessa, Umberto Tarantino, Antonella Toselli, Giovanni Carlo Isaia

**Affiliations:** 1 SIMI, (Italian Society of Internal Medicine), Rome, Italy; 2 SIE (Italian Society of Endocrinology), Rome, Italy; 3 SIMFER (Italian Society of Physical and Rehabilitation Medicine), Rome, Italy; 4 SIR (Italian Society of Rheumatology), Milan, Italy; 5 SIOMMMS (Italian Society for Osteoporosis, Mineral Metabolism and Bone Diseases), Rome, Italy; 6 SIGG (Italian Society of Gerontology and Geriatrics), Firenze, Italy; 7 SIMG (Italian Society of General Medicine and of Primary Care), Firenze, Italy; 8 SIOT (Italian Society of Orthopaedics), Genoa, Italy

**Keywords:** Osteoporosis, Fractures, Therapy

## Abstract

The purpose of this document, a result of the harmonisation and revision of Guidelines published separately by the SIMFER, SIOMMMS/SIR, and SIOT associations, is to provide practical indications based on specific levels of evidence and various grades of recommendations, drawn from available literature, for the management of osteoporosis and for the diagnosis, prevention, and treatment of fragility fractures. These indications were discussed and formally approved by the delegates of the Italian Scientific Associations involved in the project (SIE, SIGG, SIMFER, SIMG, SIMI, SIOMMMS, SIR, and SIOT).


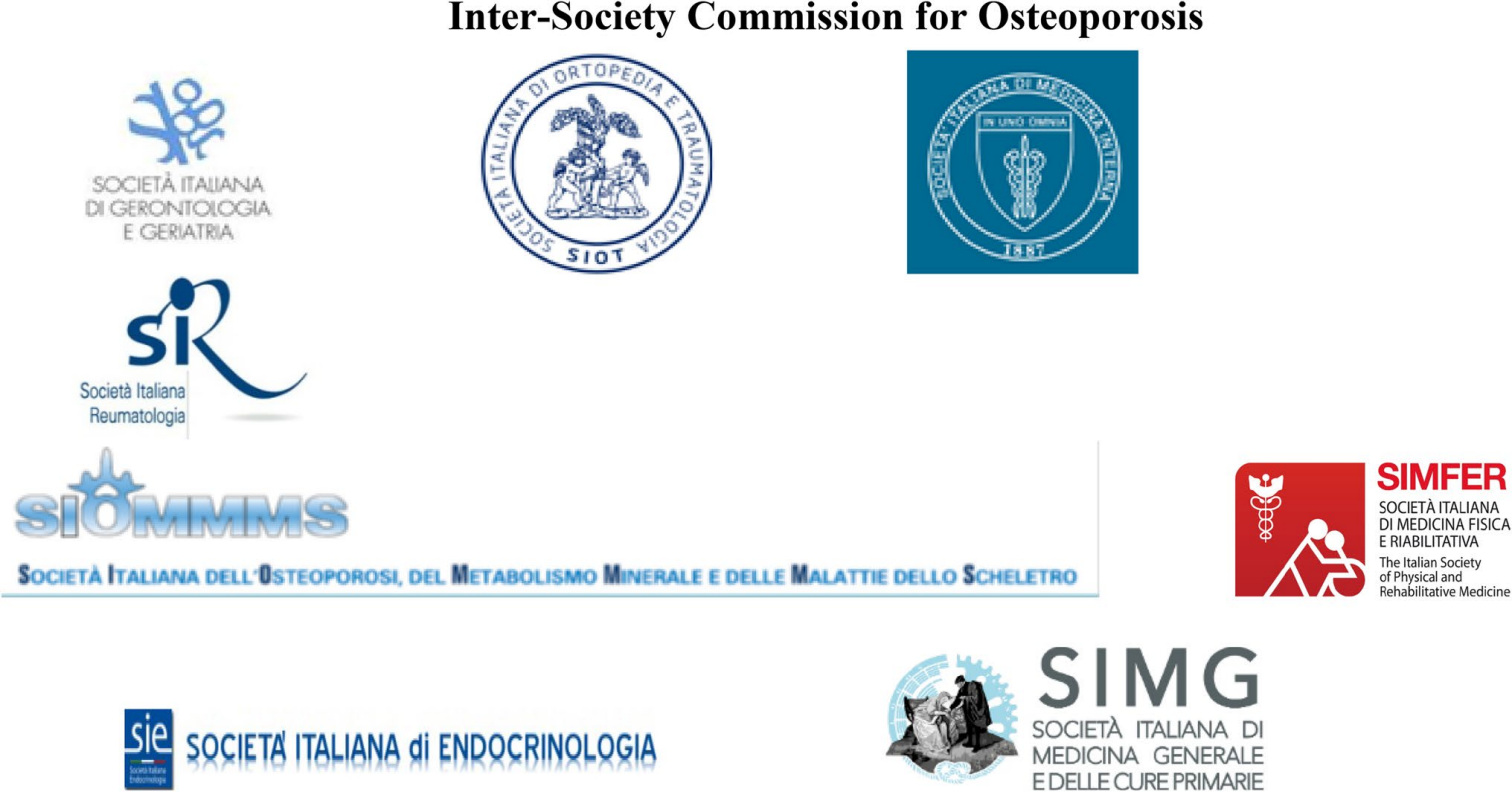


## Definition

Osteoporosis is a systemic skeletal disease characterized by a reduction in bone mass and qualitative skeletal changes (macro- and microarchitecture, material properties, geometry, and micro-damage) that cause an increase in bone fragility and higher fracture risk. There are two forms of the disease: (a) primary osteoporosis, which includes juvenile, postmenopausal, and male and senile osteoporosis; and (b) secondary osteoporosis, which is caused by a large number of diseases and medications.

Fragility fractures may occur in almost all skeletal segments, but the preferential locations are the vertebral column, the proximal ends of the femur and humerus, and the distal end of the radius (Colles fracture). Trauma due to a fall is by far the most frequent cause of fractures affecting long bones (femur, humerus, and radius), while it is more difficult to determine the cause and the exact time of fragility fractures of the vertebral body, which often go undiagnosed.

During patient evaluation, there are some clinical history details that can suggest a vertebral fracture: recent trauma, prolonged use of corticosteroids, age, structural spinal deformity, loss of height > 6 cm, and a distance between the last rib and the iliac crest < 2 fingers. It is, therefore, advisable to carefully evaluate the presence of dorso-lumbar pain, progressive loss of height, or dorsal kyphosis, which may result in alterations of the respiratory or gastrointestinal functions.

### Primary osteoporosis

- Juvenile osteoporosis

The expression juvenile osteoporosis is commonly used to indicate a form of osteoporosis found in childhood and adolescence: this disease is mostly due to genetic mutations that can lead to quantitative or qualitative alterations in the connective tissue component of bone (as in *osteogenesis imperfecta*, which is also characterized by extra-skeletal alterations), or to an altered osteoblastic activity with the particular involvement of the trabecular bone (as in the autosomal dominant form caused by inappropriate activation of the Wnt-β catenin signal). It can also be secondary to leukaemia, prolonged immobilisation, or chronic inflammatory diseases; or it can be due to the chronic administration of drugs such as anti-epileptics and glucocorticoids. When it is not possible to identify possible causes of bone loss and fragility fractures, this condition is referred to as juvenile idiopathic osteoporosis.

In accordance with the Pediatric Official Positions of the International Society for Clinical Densitometry (ISCD), the diagnosis of osteoporosis in childhood is made on the basis of a history of one or more vertebral fragility fractures, or of a history of at least two fractures of the long bones before the age of 10, or of three or more long bone fractures before the age of 19 in the absence of local pathologies, high-energy trauma, and bone mineral density (BMD) *Z*-score ≤ 2.0 standard deviation (SD) at the lumbar spine or total body less head (TBLH) scans.

(b) Postmenopausal osteoporosis

Postmenopausal osteoporosis is the most frequent primary form of the pathology, and is due to oestrogen deficiency associated with menopause, which provokes an acceleration of bone loss due to age. It is characterized by rapid loss of trabecular bone mass with perforation of the trabecular bone, while cortical bone is partially spared. This loss is responsible for fragility fractures due to load bearing, especially by the vertebrae and the distal radius. It is also generally characterized by a high bone turnover rate, with bone marrow expansion, and a prevalence of increased endosteal resorption, and also by inhibition of periosteal bone formation. BMD as determined by dual-X-ray absorptiometry (DXA) is unanimously considered to be the most important predictor of osteoporotic fractures, and is indicated, according to Italian Ministerial Decree regulating Essential Assistance Levels (EAL), in women of any age, in the presence of a major risk factor (for example, previous fragility fracture caused by minimal trauma, maternal family history of osteoporotic fracture at less than 75 years of age, menopause before 45 years of age, body mass index (BMI) < 19 kg/m^2^, and prolonged glucocorticoid therapy) and, for postmenopausal women only, the presence of at least three or more of the following minor risk factors:

1. Age greater than 65 years
2. Family history of severe osteoporosis
3. Premenopausal amenorrhoea for a period greater than 6 months
4. Inadequate calcium intake (< 1200 mg/day)
5. Smoking > 20 cigarettes/day
6. Alcoholism (> 60 g/day)
7. Male osteoporosis.

Osteoporosis is a major public health problem for men, as well; in fact, more than 20% of all hip fractures occur in males, and the incidence of vertebral fractures is about half that reported in women. Male osteoporosis is frequently secondary (about two-thirds of cases in males versus one-third in females), so it is always advisable to exclude other pathological conditions associated with osteoporosis (Table 1). Moreover, in men, the BMD DXA technique is the method of choice to determine fracture risk, and it is indicated, according to EAL, at any age, if there is a major risk factor (for example, fragility fracture, prolonged steroid therapy) or in the presence of three or more of the following minor risk factors for men over the age of 60 years:

**Table 1:** Causes of secondary osteoporosis

Endocrine or metabolic conditions	Rheumatic conditions
Hyperparathyroidism	Rheumatoid arthritis
Hypogonadism	LES
Thyrotoxicosis	Ankylosing spondylitis
Hyperadrenocorticism	Psoriatic arthritis
Diabetes mellitus	Scleroderma
Hyperprolactinaemia	Renal conditions
GH deficit	Chronic renal failure
Acromegaly	Idiopathic hypercalciuria
Blood conditions	Renal tubular acidosis
Leukaemia	Other conditions
Multiple myeloma	Anorexia nervosa
Systemic mastocytosis	Cystic fibrosis
Thalassemia	COPD
Gastrointestinal conditions	Parkinson’s disease
Celiac disease	Multiple sclerosis
Gastrectomy and gastric bypass	Drug-induced
Intestinal malabsorption	Glucocorticoids
Inflammatory bowel disease	l-Thyroxin suppressive therapy
Chronic liver disease	Heparin and oral
Primary biliary cirrhosis	Anticoagulants (AVK)
Genetic conditions	Anticonvulsants
Osteogenesis imperfecta	Aromatase inhibitors
Ehler–Danlos syndrome	Anti-androgens
Gaucher’s disease	GnRH antagonists
Glycogen storage disease	Immunosuppressives
Hypophosphatemia	Anti-retrovirals
Hemochromatosis	Thiazolidinediones
Homocystinuria	Proton pump inhibitors
Cystic fibrosis	Selective serotonin
Marfan syndrome	Re-uptake inhibitors (SSRI)

1. Family history of severe osteoporosis
2. Underweight (BMI < 19 kg/m^2^)
3. Inadequate calcium intake (< 1200 mg/day)
4. Smoking > 20 cigarettes/day
5. Alcoholism (> 60 g/day).

Although densitometric criteria for the diagnosis of osteoporosis in males are not based on levels of evidence similar to those for females, currently, the accepted diagnostic densitometric cutoff for the definition of male osteoporosis is a *T*-score < − 2.5 SD compared to young adult male subjects [1–4].

### Secondary osteoporosis

Primary osteoporosis should always be distinguished from forms of secondary osteoporosis (Table 1).

Due to special diagnostic and therapeutic implications closely related to secondary osteoporosis management, we will provide herein indications for some of the most typical or frequent forms of this condition.

- *Glucocorticoid-induced osteoporosis* Chronic exposure to glucocorticoids, both due to increased endogenous synthesis (Cushing’s syndrome), and to exogenous intake (treatment of inflammatory or autoimmune diseases), is an important cause of osteoporosis and fractures. Glucocorticoids, in fact, stimulate resorption and, above all, reduce bone formation by inhibiting osteoblast proliferation and differentiation, and promoting osteoblast and osteocyte apoptosis. The loss of bone mass caused by glucocorticoids begins early, and is more pronounced during the first 6–12 months, especially at the level of the trabecular bone (vertebral fractures, in particular, may occur early after the beginning of steroid therapy). Fragility fractures occur in between 30 and 50% of patients within the first 5 years of chronic glucocorticoid therapy, and their probability is further increased if other risk factors are present, such as old age, previous fractures and, in women, menopause. The incidence of fractures is related to the dose and duration of glucocorticoid therapy, and is also influenced by the underlying disease for which it was prescribed (e.g., rheumatoid arthritis and inflammatory bowel disease). Although lower doses are less harmful than higher ones, a threshold below which no bone damage occurs is controversial. The negative impact on bone health exerted by glucocorticoids administered by inhalation is still a very controversial topic: undoubtedly, their use is much less harmful to bone, in contrast to systemic administration, although doses > 800 mcg/day of budesonide (or equivalent), especially if prolonged, may be associated with accelerated loss of bone mass and increased risk of fractures. In glucocorticoid-induced osteoporosis, the risk of fractures is much higher than could be expected based on the patient’s densitometric values, and decreases rapidly after discontinuation of treatment.
- *Organ transplant osteoporosis* The estimated prevalence of fragility fractures is approximately 10–15% in patients waiting for solid organ transplants (kidney, heart, liver, and lung), due to the negative effects of the underlying condition on bone tissue. After transplant, the percentage of patients with osteoporosis increases dramatically. Bone loss is greatest in the first year after surgery, but can also persist, albeit at a slower pace, during subsequent periods. During the first 3 years after a transplant, the percentage of vertebral fractures due to bone fragility reaches a peak, and affects approximately 30–40% of patients. The main fracture-inducing factor is the immunosuppressive therapy, in particular, the use of cortisone, which is initially administered at very high doses, and, in the majority of patients, for an indefinite period; other relevant risk factors common to all types of transplants (at least in the long term) are greater age and female gender. Even the intrinsic factors relating to organ disease can be involved in the development of bone fragility: the most representative example of this specific form of osteoporosis is persistent, very long-term severe forms of secondary hyperparathyroidism, which can affect up to 50% of patients after a kidney transplant, even when the transplant is functional.
- *Drug osteoporosis* Many types of drugs are associated with osteoporosis and fragility fractures. Many of these associations are derived from data obtained from epidemiological and retrospective studies, and in many cases, the incidence level is quite low. In addition to steroid therapy, it is now well known that aromatase inhibitors and GnRH are associated with increased risk of fragility fractures. A significantly increased risk of vertebral fractures and hip fractures has been associated with the use of proton pump inhibitors (PPI), especially if used for more than 12 months. In the case of serotonin re-uptake inhibitors (SSRIs), association with hip fracture appears within the first year of use of this drug in both genders, especially in those patients over-70. A retrospective study demonstrates that in patients adhering to alendronate treatment, the combination with SSRIs is accompanied by a higher risk of major osteoporotic fractures.
  Levothyroxine (when administered in suppressive doses) is associated with an increased risk of fracture. The use of pioglitazone and rosiglitazone is strongly associated with a significant increased (three to fourfold) risk of fracture of the hip and humerus in postmenopausal women. There is extensive literature about the association between the use of some first-generation anticonvulsants (carbamazepine, phenobarbital, and phenytoin) in epileptic patients, especially if used in polytherapy, and low bone mass, while the risk of hip fracture increases from 2 to 6 times. Long-term use of unfractionated heparin leads to an increased risk of fracture (+ 2.5 to 5%), while there are no data about low-molecular-weight heparin use. On the other hand, the risk of fractures due to warfarin is controversial [5–10].

## Epidemiological remarks

The epidemiological impact of osteoporosis is impressive. In Italy, about 3.5 million women and 1 million men suffer from osteoporosis, and, over the next 25 years, the percentage of the over-65 population will increase by 25%, so a proportional increase of this condition is to be expected. In the over-50 population, the number of hip fractures exceeds 90,000, and in 2010, more than 70,000 vertebral fractures were reported by emergency services, but considering that many of these fractures go undiagnosed, it is believed that the actual figure is at least ten times greater.

It should be remembered that osteoporotic fractures of the hip and spine increase the relative risk of mortality. For hip fractures, it is about 5–8 times greater in the first 3 months after the event, decreasing over the following 2 years, but remains high at the 10-year follow-up; in absolute terms the incidence is up to 9% at 1 month after the event, and 36% at 1 year, substantially comparable to stroke and breast cancer and four times greater than for endometrial carcinoma. Moreover, 50% of women with hip fracture suffer from a substantial reduction in their level of self-sufficiency which, in approximately 20% of cases, involves long-term institutionalisation.

Colles’ fracture is also an early and sensitive marker of skeletal fragility, predisposing the patient to additional fractures, in particular of the hip.

The economic implications of such a widespread disease are naturally very important: it is estimated that, in Italy, the cost of treatment of osteoporotic fractures is greater than 7 billion Euros per year, of which “only” 360,000 are for secondary drug prevention. Proximal femur fractures, in particular, contribute to 60% of total costs, vertebral fractures for 4%, wrist for 1%, while the remaining 35% is by other fractures. To this, of course, must be added the cost of pharmacological therapies and social spending (work days lost, disability, etc.).

Fragility fractures cause complex disability, significant morbidity, reduction in quality of life, and functional limitations. A patient with osteoporosis requires comprehensive care, multi- and interdisciplinary intervention, and an individual rehabilitation plan consisting of programmes oriented towards specific areas of intervention. Based on the International Classification of Functioning, Disability and Health (ICF), the typical spectrum of functional problems experienced by subjects with osteoporosis (osteo-metabolic balance, motor function, posture, balance, coordination, mobility, gait, and quality of life) have been defined. The most relevant ICF categories for osteoporotic patients have recently been defined and implemented in a specific “ICF Core Set for Osteoporosis” [11–15].

## Risk factors

Fracture pathogenesis must take into account the many factors that influence both bone strength as well as frequency and type of trauma. Risk of osteoporotic fracture is determined by a combination of factors that act mainly through a reduction of BMD, factors that are partially or totally independent of BMD (bone tissue characteristics) and extra-osseous factors that cannot be evaluated by means of BMD. The distinction is obviously not inflexible, and several risk factors act simultaneously through multiple mechanisms. In patients with multiple risk factors, fracture risk is higher than in patients with a single risk factor, including an isolated reduction in BMD. As a result, the determination of BMD can adequately diagnose osteoporosis (diagnostic threshold), while the identification of high fracture risk patients needing specific drug treatment (therapeutic threshold) requires an evaluation of combined BMD and independent risk factors.

- *Age* The incidence of osteoporotic fractures increases exponentially with age. The risk of fracture associated with advancing age is only partially due to BMD reduction, and depends largely on other factors, such as qualitative alterations in bone structure, increase in the frequency of falls, and slowdown of protective responses. Thus, for a given BMD, fracture risk is higher in older people than in younger people.
- *Family history of fragility fractures* A family history of fractures, especially of the femur, influences fracture risk independently of BMD, and is the most valuable prognostic indicator.
- *Previous fractures* In both genders, the previous fragility fractures are an important risk factor for subsequent fractures, irrespective of BMD. All previous non-traumatic fractures increase the risk of new fractures, although, to varying degrees, also depending on their location and number. Special prognostic relevance is given to vertebral (including morphometric fractures), wrist, femur, and humerus fractures. Subjects with three or more vertebral fractures, risk new fractures almost ten times more than those who do not experience similar previous events, and 2–3 times more than those who have only one fracture. As regards mild vertebral fractures, these represent a risk factor for more vertebral fractures, while their negative prognostic significance regarding non-vertebral fractures is uncertain.
- *BMD* BMD reduction is a significant risk factor for fractures: this depends on peak bone mass attained at the height of bone development, and bone loss related to menopause and ageing, and is influenced by genetic and nutritional factors, lifestyle, behaviour, various diseases, and drug treatment.
  Numerous prospective epidemiological studies carried out mostly by measuring BMD using the DXA technique at axial locations (femoral neck, total hip, and lumbar spine) have ascertained that any reduction in BMD SD increases the risk of fracture 1.5–3 times.
- *Smoking* Smoking (cigarettes in particular) is an independent risk factor for vertebral and appendicular fractures.
- *Immobility* Is considered a moderate risk factor.
- *Comorbidities* Many pathological conditions are associated with increased rates of fracture risk. In many of these conditions, it is believed that the risk is mediated by BMD reduction. However, comorbidities often involve different mechanisms, including chronic inflammation, altered bone quality, impairment of general health conditions, specific complications, decreased mobility, decreased muscle mass and muscle function (sarcopenia), increased risk of falling, and vitamin D deficiency, which is very frequent in Italy, especially in the elderly population. The diseases most frequently associated with an increased risk of fracture are: rheumatoid arthritis, inflammatory bowel diseases, untreated hypogonadism (GH deficiency, oophorectomy or bilateral orchiectomy, androgen deprivation in men with prostate cancer, chemotherapy, or adjuvant hormonal therapy in women with breast cancer), organ transplants, COPD, diabetes mellitus types 1 and 2, disabling motor diseases, and prolonged immobility (Parkinson’s disease, stroke, muscular dystrophy, and spinal cord injury).
- *Risk factors for falls* These are of fundamental importance, especially in older individuals. The most important of these are deafness, visual disorders, neuromuscular disorders, Parkinson’s disease, dementia, malnutrition, alcoholism, and vitamin D deficiency. Environmental factors capable of promoting falls such as physical barriers, carpets, slippery floors, poor lighting environments, etc. must also be corrected.

### Overall assessment of fracture risk

Using specific algorithms, it is possible to perform an integrated assessment of BMD including the most important risk factors, partially or wholly independent of BMD, so as to arrive at a more accurate estimate of middling-term (5–10 years) risk of fragility fractures, and, therefore, identification of subjects in whom drug treatment is the most appropriate therapeutic solution.

The definition of clinical risk factors independent of BMD included in these algorithms has been considered in a series of studies and meta-analyses that have identified their importance, and also their ease of identification and quantification. The greater importance of some BMD independent clinical risk factors (diabetes mellitus, androgen deprivation therapy, and use of aromatase inhibitors) has also resulted, in the long run, in their being significantly more considered when establishing criteria for the reimbursement of drug costs in cases of osteoporosis in Italy (Note 79, AIFA). Currently, to evaluate multiple risk-factor combinations, it is possible to use mathematical algorithms that quantify risk in terms of “10-year fracture risk.” One of the algorithms most commonly used today is FRAX^®^ (http://www.shef.ac.uk/FRAX/), which, however, has inherent limitations, due mainly to the use of dichotomous variables only. In Italy, to improve the accuracy of FRAX^®^, it was converted into a version known as “Derived Fracture Risk Assessment” or DeFRA (http://defra-osteoporosi.it). It only provides an estimate of risk similar to FRAX^®^ on the basis of continuous variables (age, BMI, BMD), but is more accurate as it evaluates other clinical risk factors in a more detailed (e.g., the location and number of previous fractures) and complete manner (e.g., other osteoporosis-inducing drugs, other comorbidities, not only femoral but spine BMD too). The data contained in Health Search, a general medicine database, containing data for about 1 million patients aged between 50 and 85, have permitted us to verify that the incidence for a 5-year period (per 1000 people/year) of osteoporotic fractures is 11.56 (95% CI 11.33–11.77) in females, and 4.91 (95% CI 4.75–5.07) in males. Predictive factors for fragility fractures prove to be in line with those provided by the FRAX^®^ algorithm, leading to the development of a present-day score system called FraHS, available to general practitioners, and, therefore, of immediate use in favour of the entire population [1, 16–23].

## Diagnosis

Diagnosis of osteoporosis and assessments of fragility fracture risks are based on case history, physical examination, laboratory, and diagnostic tests.

Case histories require the collection of information related to patients’ medical histories, lifestyle, and appropriate assessment of risk factors. Of particular importance is the history of previous fragility fractures and family history of fractures. It is common knowledge that a history of femur fractures in parents significantly increases the risk of hip fractures, and, to a lesser extent, of all osteoporotic fractures, in their offspring. Finally, the presence of comorbidities should be carefully investigated, any medication that may interfere with bone metabolism and, in women, their gynaecological history, and the age of the onset of menopause are also significant.

A physical examination should evaluate the patient’s posture, especially if there is an increase in kyphosis or a decrease in height that may indicate the presence of one or more vertebral deformities.

### Diagnostic imaging

Diagnostic imaging of osteoporosis and fragility fractures includes evaluation of BMD using DXA, quantitative computerized tomography (QCT) or ultrasound (QUS) studies, and conventional radiology to diagnose spinal fractures.

- Computerized X-ray bone densitometry

X-ray densitometry (DXA) makes it possible to measure bone mass and bone mineral density (BMD) in g/cm^2^ of projected bone area accurately and precisely.

According to the WHO, densitometric diagnosis of osteoporosis is based on technical DXA evaluation of mineral density, to be compared to the average of healthy adults of the same gender (peak bone mass). The unit of measurement is represented by SD from the mean bone mass peak (*T*-score). BMD can also be expressed by means of comparison to average values for subjects of the same age and gender (*Z*-score). It has been observed that risk of fracture begins to increase exponentially with densitometric *T*-score values of < − 2.5 SD that, according to the WHO, represent the threshold level by which to diagnose the presence of osteoporosis. Bone densitometry represents, therefore, a diagnostic test for osteoporosis and risk of fracture, just as blood pressure measurement is used to diagnose hypertension and, therefore, the risk of having a stroke. According to the WHO, when interpreting the results of BMD, the following definitions should be used:

1. Normal BMD is defined by a *T*-score between 2.5 and − 1.0 (therefore, patient BMD lies at between 2.5 SD above the mean and 1 SD below the mean for a healthy young adult of the same sex).
2. Osteopaenia (low BMD) is defined by a *T*-score of between − 1.0 and − 2.5 SD.
3. Osteoporosis is defined by a *T*-score equal to or less than − 2.5 SD.
4. Severe (or established) osteoporosis is defined by a *T*-score below − 2.5 SD and by the simultaneous presence of one or more fragility fractures.

A densitometric assay is considered the best predictor of osteoporotic fracture risks, although it should be noted that diagnosis of osteoporosis could not be established on the basis of densitometry alone, but always require adequate clinical evaluation.

The *T*-score diagnostic threshold, moreover, does not coincide with the therapeutic threshold, since other factors, skeletal and extra-skeletal alike, influence both the risk of fracture for individual subjects, and the decision whether or not to undertake pharmacological treatment.

Since most clinically relevant osteoporotic fractures occur at vertebral and femoral level, the most frequently measured sites are the lumbar spine and proximal femur. Densitometric examinations can be carried out at the level of the lumbar spine (L1–L4), of the total hip and the femoral neck alone. The lowest *T*-score value for these three sites is considered the densitometry result.

The accuracy of densitometric results may be reduced by the possible presence of interfering factors that need to be taken into due consideration by those who refer to or perform this measurement. For example, a fractured vertebra or one with postarthritic focal accumulation must be excluded from the densitometric analysis, and at least two adjacent lumbar vertebrae evaluated. For this reason, lumbar densitometry is often inaccurate after the age of 65 due to interference of osteoarthritis signs, extra-skeletal calcifications, or vertebral fractures; therefore, after this age, it is preferable to assess femoral densitometry.

Peripheral measurements of the forearm are reserved for special circumstances and in particular for patients in whom lumbar or femoral evaluation is not possible, or not accurate, or if they are severely obese, or suffer from primary hyperparathyroidism.

Recently, DXA software has been developed, which, in addition to densitometry, makes it possible to evaluate a number of geometric parameters related to bone strength, such as HSA (hip structural analysis) and TBS (trabecular bone score). HSA evaluates strength indices and geometric parameters of the proximal femur. Of these, the most significant are cross-sectional area, cross-sectional moment of inertia, section modulus, and buckling ratio. TBS is a software that processes degrees of inhomogeneity in spinal densitometry scans, while providing indirect information on trabecular microarchitecture. Hitherto published studies show that TBS improves, compared to BMD measurement alone, the ability to predict fracture risks. It seems to play a particularly significant role in the classification of those at risk for fragility fracture with BMD values within the normal or osteopaenia range. This application is approved by the FDA, but its usefulness in clinical practice has not been clearly defined.

The Italian Ministerial Decree regulating Essential Assistance Levels (EAL) considers risk factors, in the presence of which densitometric investigation is indicated [3, 24–27].

(b) Bone ultrasound

Ultrasound studies (Quantitative US, QUS) provide two parameters (speed and attenuation) that are indirect indices of bone mass and structural integrity, and are measured mainly at two sites, the phalanges of the hand and the calcaneus. It has been demonstrated that ultrasound parameters used to predict the risk of osteoporotic fractures (vertebral and femoral) are not inferior to lumbar or femoral DXA, both in postmenopausal women and in men. This technique does not represent a direct measurement of bone density, and therefore, discordant results between QUS and DXA do not necessarily indicate an error, but, rather, that the QUS parameters are independent predictors of fracture risk influenced by other characteristics of bone tissue. Moreover, for this reason, QUS cannot be used for the diagnosis of osteoporosis according to WHO criteria (*T*-score < − 2.5 SD). An important limitation of QUS is represented by the heterogeneity of the devices that provide values not always related to each other; however, it can be useful when it is not possible to perform a lumbar or femoral DXA, and may be recommended for epidemiological investigations and the first-level screening, considering its relatively low costs, easy portability, and the absence of radiation. In general, a reduced ultrasound value, in the presence of other clinical fracture risk factors, can justify therapeutic intervention, while a high ultrasound value, in the absence of risk factors, indicates unlikely probability of osteoporotic fractures, and, therefore, the inutility of further investigation.

(c) Conventional radiology

**Fig. 1:**
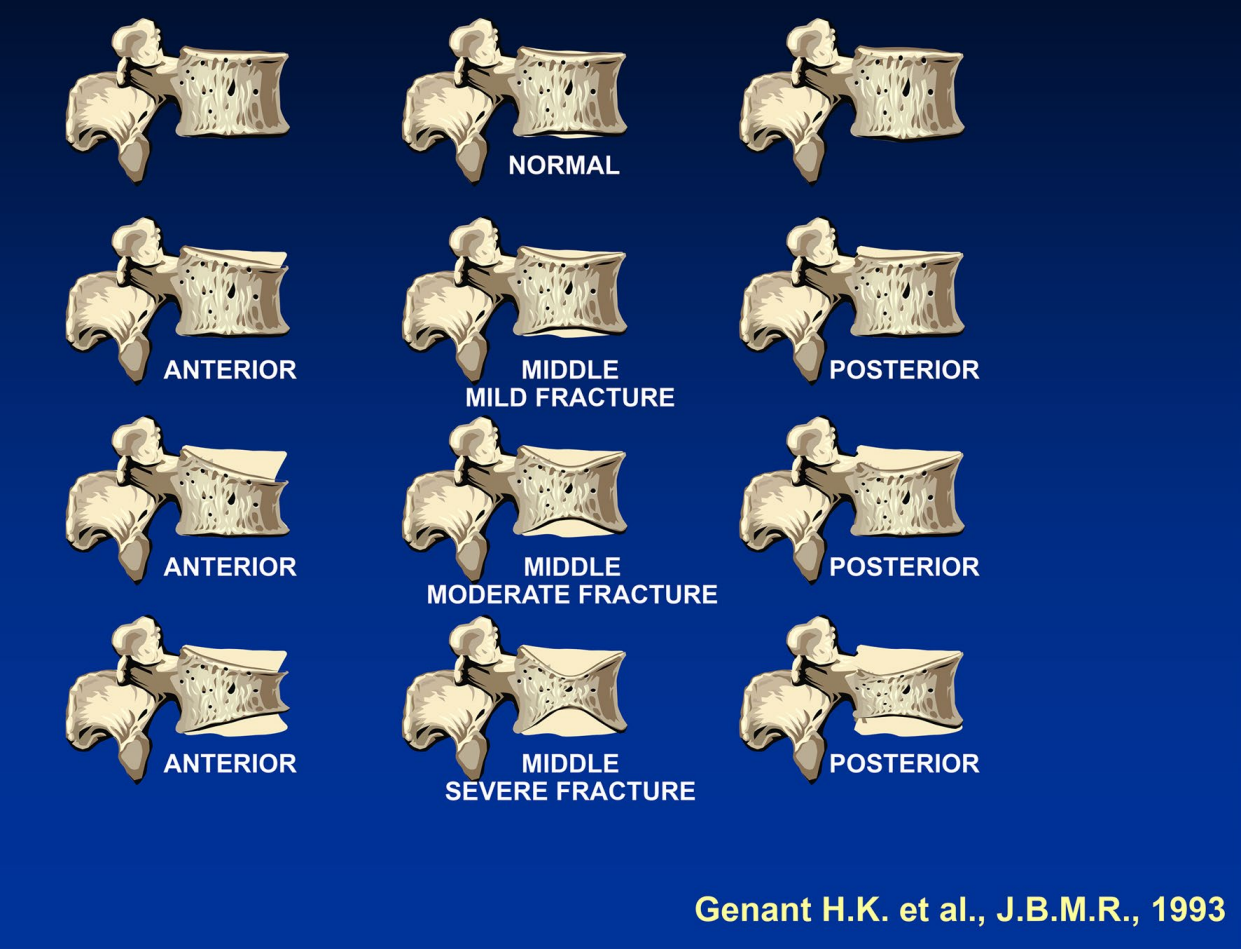
Evaluation of spinal deformities based on Genant criteria

*Traditional radiology* This makes it possible to diagnose osteoporosis fractures in the most commonly involved sites (spine, ribs, pelvis, proximal femur, proximal humerus, distal radius, and calcaneus). In particular, radiological studies and semi-quantitative or quantitative vertebral morphometry allow the identification and correct classification of vertebral deformities that do not correspond in all cases to vertebral fractures due to bone fragility. X-ray studies, depending on the type and severity of spinal height reduction, make it possible to identify three types of vertebral fractures: wedge-shaped (anterior), biconcave (middle), and total vertebral collapse. To arrive at a more accurate identification, other methods of assessment exist, providing more or less quantitative analyses of spine deformation. These methods may be divided into two classes: (a) semi-quantitative (SQ) visual methods and (b) quantitative morphometric methods. The SQ methods are based on an initial phase of visual evaluation of images of the spine for a differential diagnosis of vertebral deformities providing; therefore, a visual gradation of osteoporotic vertebral fractures considered mild, moderate, or severe (the Genant criteria) (Fig. 1).

*Vertebral morphometry* This is a quantitative method for the diagnosis of vertebral fractures based on the measurement of vertebral height, and is carried out on the images of lateral projections of the thoraco-lumbar spine, performed by the conventional radiology (MRX) or with DXA (MXA), using VFA software (vertebral fracture assessment) that makes it possible, using radiation doses (50 µSv, about 1/100 lower compared to conventional radiography) to capture the entire dorsal and lumbar spine in a single image while providing contextual measurements of vertebral body height limited to the T4–L4 area. The vertebral morphometry technique is applied to images to assess the severity of vertebral fractures previously diagnosed by means of SQ, and to evaluate the possible occurrence of new fractures or a worsening of preexisting fractures during patient follow-up. However, vertebral morphometry cannot be performed separately from a previous qualitative X-ray analysis to rule out deformity due to causes other than osteoporosis.

d. Spinal MRI

The use of MRI in instrumental diagnoses of vertebral fragility fractures is indicated when several vertebrae are involved, because it makes it possible to determine- on the basis of the presence of signal changes in T2 and STIR, of bone oedema—to distinguish recent fractures from older ones, and to identify vertebrae, not yet deformed, presenting signs of impending structural failure.

e. Spinal CT

Using vertebral CT, it is possible to study the bone component of the fractured vertebra in detail, and to obtain information, for example, possible dislocation of bone fragments into the medullary canal in cases of traumatic fracture. CT is not indicated in routine evaluations of osteoporosis, but can be a useful investigation complementary to MRI in some cases [28–34].

### Laboratory diagnosis

The first and second-level laboratory test**s** (Table 2) play a key role in the diagnosis of osteoporosis, inasmuch as they:

**Table 2:** Levels I and II laboratory tests

First-level tests	Level II tests
ESR	Ionised calcium
Complete blood count	Thyroid stimulating hormone (TSH)
Total protein + protein electrophoresis	Parathyroid hormone (PTH)
Serum-calcium levels^a^	25-OH-vitamin D
Phosphoraemia	Cortisol after overnight suppression test with 1 mg of dexamethasone
Total alkaline phosphatase	Free Androgen Index (in males)
Creatininaemia	Serum and urine immunofixation
24 h urinary calcium	Antitransglutaminase antibodies
	Specific tests for associated diseases (e.g., % ferritin and transferrin saturation, tryptase, etc.)

^a^Corrected serum-calcium (mg/dL): total serum-calcium levels (mg/dL) + 0.8 [4 − albumin in g/dL]

1. permit differential diagnoses, with other metabolic diseases of the skeleton, that may result in a reduced BMD;
2. may make it possible to diagnose forms of secondary osteoporosis;
3. can help to guide pharmacological choices and provide useful elements for evaluating adherence to therapy

First-level tests are key elements in the diagnosis of osteoporosis. In fact, if they are normal, it is possible to exclude; in 90% of cases, other metabolic diseases of the skeleton or forms of secondary osteoporosis. Second-level tests are crucial when seeking to identify secondary forms of osteoporosis, and their choice must be based on the medical history and clinical evaluations of individual patients.

#### Bone turnover markers

Bone turnover markers are mainly used to obtain information about the extent of new-bone-formation and resorption processes. They are overall indicators of skeletal remodelling, and, therefore, vary considerably at analytical and biological level: therefore, there is no indication for their use in routine evaluations of individual patients. In population studies, especially in postmenopausal women, they may prove useful when seeking to estimate the risk of fracture, irrespective of BMD. They have also been used widely in clinical trials aimed at monitoring the efficacy and mechanism of action of new drugs. Those commonly used in the assessment of bone neoformation are osteocalcin, bone isoenzyme of alkaline phosphatase (B-ALP), and type I collagen propeptides (PINP and PICP), while the most common markers of resorption are urinary pyridinoline (PYR), urinary deoxypyridinoline (DPYR), and serum levels of type I collagen telopeptides (NTx, CTx). Their significant alteration makes it possible to orient diagnosis towards primary or secondary diseases typical of the skeleton (Paget’s disease of bone, osteomalacia, hypophosphatasia, bone metastases, etc.). Because it is possible to find significant changes in markers after a few weeks after beginning the treatment, it has been proposed that they be used also to evaluate patient adherence to drug treatment.

#### Genetic evaluation

Polymorphism of genes encoding collagen type 1 (COLIA1), oestrogen (ER), and vitamin D (VDR) receptors has been proposed as possible genetic determinants of the risk of osteoporosis. Each of these polymorphisms only accounts for less than 30% of the variance found in bone mass and even less than that when it comes to risk of fracture. Therefore, routine screening of genetic polymorphisms is not indicated either for fracture risk assessment or for determining therapeutic choices. Genetic analysis is, however, recommended in those rare cases where clinical and laboratory tests suggest a monogenic bone disease (e.g., hypophosphatasia, Gaucher disease, and juvenile osteoporosis due to COL1A1 mutations) [23, 35, 36].

## Non-pharmacological measures for osteoporosis prevention and treatment

Osteoporosis prevention consists of using measures to prevent or slow down the onset of the disease. Treatment is directed, instead, to subjects already suffering from osteoporosis, with or without preexisting fractures but with a high first-fracture or further fragility fracture risks. Prevention is first implemented and generally consists in the correction of risk factors. Non-pharmacological intervention and elimination of modifiable risk factors (smoking, alcohol abuse, and environmental risks of falls) should be recommended for all.

### Nutritional approach

#### Calcium

An adequate intake of calcium increases the density of the bone matrix in children and adolescents, maintains it in adults, and slows down its loss in women after menopause. The main source of calcium is milk and its derivatives, and, to a lesser extent, nuts (almonds), some vegetables (cabbage, spinach, and turnips) and pulses. The average calcium intake in the Italian population is insufficient, especially in the elderly. This dietary deficiency may contribute to negative calcium balance and to secondary hyperparathyroidism. Daily calcium requirements depend on age and certain conditions (Table 3).

**Table 3:** Calcium requirements at different ages and under different conditions

Calcium requirements	mg/day
1–5 years	800
6–10 years	800–1200
11–24 years	1200–1500
25–50 years	1000
Pregnant or nursing	1200–1500
Postmenopausal women receiving oestrogen/men 50–65 years of age	1000
Postmenopausal women without oestrogen treatment/men aged > 65 years of age	1200

Supplemental calcium is especially indicated during pregnancy and lactation. Supplementation of calcium intake and calcium supplements in the diet, and this intervention alone, has been shown to produce modest increases in densitometry in women with deficient dietary intakes and in women 5 years after the onset of menopause. It has been reported that the sole administration of calcium does not produce a complete, but only a slight reduction in fracture risk, particularly in the elderly, but the most convincing documentation of its anti-fracture efficacy has been shown when it is administered in combination with vitamin D. The efficacy of an adequate calcium intake, as well as vitamin D, is proportional to the severity and the frequency of the deficiencies in the population examined.

The risk of non-oxalic kidney stones can increase with the intake of calcium supplements, which is reduced with a diet rich in calcium, and the safety of calcium supplements is questioned as regards possible increases in vascular calcification and cardiovascular risk: although the most recent publications have not confirmed correlations between calcium intake and cardiovascular diseases, it is recommended that calcium supplementation adheres to the following guidelines:

1. Always estimate diet calcium intake by means of a brief questionnaire before prescription;
2. Always try to ensure an adequate intake of calcium from food and water rich in calcium;
3. Use dietary supplements only when calcium assumption is insufficient, indicating intake at meals and the minimum dose necessary to satisfy requirements, possibly dividing intake into a number of doses (for example, 500 mg at lunch and 500 mg at dinner) [37–44].

#### Vitamin D

Vitamin D is contained almost exclusively in animal fats, fish, liver, milk, and dairy products, while the amount of vitamin D in some vegetable fats is negligible; approximately 20% of circulating vitamin D derives from food, while it is largely produced by endogenous synthesis in the skin following exposure to UVB sun rays, a process increasingly less efficient with advancing age. Consequently, there is a frequent need for supplementation, especially in old age, with vitamin D (cholecalciferol or ergocalciferol, namely D3 or D2), which, if associated with an adequate intake of calcium, has proved useful in the primary prevention of fractures, in the elderly.

The effects of vitamin D supplementation on BMD are modest on average, proportional to the degree of deficiency and documented mainly only as regards the femur. The anti-fracture effect of vitamin D is modest and documented, not for vertebral, but only for hip and non-vertebral fractures, and seems to be mediated also by a reported reduction in the risk of falling; in all cases, adequate calcium and vitamin D are a prerequisite for all specific drug treatment, because the lack of calcium or vitamin D is one of the most common causes of failure or reduced response to drug therapy for osteoporosis. A slight but significant reduction in mortality in the elderly associated with the use of cholecalciferol has also been reported, but there is currently no evidence of extra-skeletal benefits, although there is a strong pathophysiological rationale for this. The current indications on how to interpret different levels of 25 (OH) D are shown in Table 4.

**Table 4:** Interpretation of plasma levels of 25 (OH) D

nmol/L	ng/mL	Interpretation
< 25	< 10	Severe deficiency
25–50	10–20	Deficiency
50–75	20–30	Insufficiency
75–125	30–50	Ideal range
125–375	50–150	Possible side effects
> 375	> 150	Intoxication

Risk conditions for hypovitaminosis D are well known, and there exists a wide therapeutic safety range regarding vitamin D supplementation, due to the regulation of physiological mechanisms of vitamin D hydroxylation. Dosage of serum levels of 25 (OH) D is considered to be the best indicator of vitamin D levels, even if, since it is not a low-cost procedure, it is not always justified from an economic point of view, especially in the elderly where hypovitaminosis D is known to be considerably widespread condition. It is, therefore, not recommended as a routine evaluation, let alone as a screening test, but must be reserved for cases of uncertainty featuring comorbidities or risk of severe hypercalcemia. If the usually recommended doses (< 4000 IU/day) are used, it is not considered essential to perform 25 (OH) D dosage, even for the purpose of monitoring. When deemed appropriate, a 25 (OH) D blood dosage can be performed to obtain a steady state (approximately 3–6 months after the start of supplementation) to check that the dose is adequate and to allow for possible dose adjustments. The objective is to reach a circulating concentration of 25 (OH) D of between 30 and 50 ng/mL (75–125 nmol/L), that is stable over time.

#### Vitamin D supplementation

Vitamin D deficiency is so common in Italy in the elderly population in general and in subjects at risk of fragility fracture, that it may be considered the rule, even without measuring plasma 25 (OH) D. When, as often happens, it is not possible to correct this deficiency through diet or with an appropriate and non-risky exposure to sunlight, it is necessary to use cholecalciferol supplements, preferably in a daily or weekly dose, avoiding the hydroxylated metabolites in position 1 (calcitriol and alfacalcidol) that overcome endogenous regulation, but may expose the patient to risk of hypercalcemia. Daily vitamin D supplementation is a more physiological approach to supplementation; however, to improve adherence to treatment, equivalent weekly or monthly dosage doses are justified from a pharmacological point of view.

If the administration of high doses (boluses) is deemed appropriate, it is recommended that these do not exceed 100,000 IU, because, at higher doses, an increase of bone resorption indices has been seen, and also a paradoxical increase in fractures and falls.

To rapidly obtain adequate serum levels of 25 (OH) D, D_3_ is preferred to D_2_, and it is better to administer this orally, limiting the use of intramuscular application to patients with severe malabsorption syndromes.

The aim of vitamin D deficiency and insufficiency therapy is to restore normal serum levels and thus of deposits of 25 (OH) D, in a brief time. The cumulative dose to be administered within a few weeks may vary depending on the severity of the deficiency and the body mass. The weekly administration of 50,000 IU of cholecalciferol during 2–3 months can restore values to normal levels in severe deficiency cases. This must be followed by a maintenance dose of up to 2,000 IU daily or equivalent doses administered weekly or monthly. These doses should be reduced accordingly if basal values are achieved, for example, or in the case of failure.

As to the use of alternative hydroxylated metabolites of vitamin D (calcifediol, 1-alpha-calcidiol, and calcitriol), there are still no adequate comparative dose-equivalent evaluations with respect to vitamin D, or documentation of anti-fracture efficacy analogous to those available for cholecalciferol’s ability to provide rationale-based indications under specific conditions. In particular: (a) calcifediol [25 (OH)D_3_], which induces a more rapid increase in levels of 25 (OH) D, due to different pharmacokinetics and a lower volume of distribution relative to cholecalciferol, may be indicated in the case of 25-hydroxylation deficits (e.g., severe liver failure, male hypogonadism, and inactivating mutations of the gene encoding enzyme 25-hydroxylase), obesity, and intestinal malabsorption; (b) calcitriol [1-25 (OH)_2_D_3_] is indicated in conditions of 1-alpha-hydroxylase deficiency (i.e., moderate-to-severe renal insufficiency, hypoparathyroidism, and mutations of the gene encoding enzyme 1-alpha-hydroxylase) and intestinal malabsorption.

The 1-hydroxylated metabolites of vitamin D can induce hypercalcaemia and hypercalciuria, which, therefore, must be checked by means of periodic monitoring of serum and urinary calcium. Even in these cases, cholecalciferol intake should be ensured in view of its known autocrine and paracrine activities and its potential extra-skeletal effects. If calcitriol and 1-α calcidiol are used, a useful contribution of cholecalciferol is ensured with a view to achieving the recommended circulating concentrations 25 (OH) D3 [45–54].

#### Other nutrients

Increases in protein consumption in patients with inadequate intake reduce the risk of hip fracture in both genders. Adequate protein intake is necessary to maintain the functions of the musculoskeletal system, but also to reduce the risk of complications after an osteoporotic fracture. In fact, an adequate protein intake (1.0–1.2 g/kg/day with at least 20–25 g of proteins per meal) associated with physical resistance exercises (muscle-strengthening exercises) increases muscle mass and strength. Even other micro-nutrients such as zinc, silicon, vitamin K, vitamin E, vitamin B6, vitamin B12, and magnesium seem to have a protective role with regards to bone and muscle.

### Physical activity

It is a well-known fact that even short periods of immobilisation adversely affect bone mass, and it is, therefore, important to maintain an appropriate level of physical activity, keeping in mind, however, that competitive physical activity in young women may lead to exaggerated hormonal and nutritional abnormalities that can be detrimental to bone.

Types of physical activity divided into two basic categories:

1. low or high impact aerobic activity (e.g., jogging, soccer, basketball, volleyball, baseball, racket sports, and gymnastics).
2. muscle-strengthening activities (weight lifting, body building, swimming, cycling or exercise bikes, and use of weights for static exercises).

One of the most common forms of aerobic exercise is walking, which is very well accepted by older people, because it is bland, can be self-managed, and easily practised. Meta-analyses, however, have highlighted the absence of significant effects of walking on lumbar and femoral BMD. In postmenopausal women, aerobic training, and, in particular, high-intensity and speed walking, interspersed with jogging, climbing stairs, and stepping, can limit reductions in bone density. Multi-component training, including moderate-to-high impact exercises, muscle strengthening, and balance exercises, has a positive effect on both the femur and the lumbar spine. Training with vibrating platforms is of dubious efficacy in improving bone density at specific sites such as the femoral neck and spine. Some epidemiological studies have shown a correlation between physical activity and lower risk of fracture.

Prescription of exercise in the elderly and osteoporotic patient should always be preceded by a thorough medical examination, which is useful to define the intensity of feasible exercise based on muscle strength, balance, gait, cardiovascular function, and comorbidities. Encouragement of even modest physical activity in the elderly can help to reduce the risk of fall and therefore of fracture. The recommendation to carry out a minimum of physical activity (walk more than 30 min a day, outdoor, if possible), despite the inadequacy of documentation attesting its benefit to bone mass, appears acceptable due to its effects on the risk of falling and, indirectly, on 25 (OH)D levels [55–64]

### Prevention of falls

Most fractures, especially of the hip, are caused by falls, and the risk factors for these (physical disabilities, balance disorders, neuromuscular disorders, visual impairment, cardiovascular disease, past medical history of falls, drug treatment, and cognitive deficits) are often modifiable in a context of multidisciplinary intervention.

Physical activity, in particular personalized muscle-strengthening exercises, balance, and gait rehabilitation, are able to reduce the risk of falls related to trauma in the elderly. Individual evaluation of fall risks and associated prevention recommendations such as a reduction in the use of psychotropic drugs has a positive impact on falls. A fall-prevention strategy for the elderly, including adequate intake of vitamin D, physical exercise, and education regarding risks within the home, is highly recommendable.

An alternative or, rather, supplementary strategy capable of reducing the risk of hip fracture is that of mitigating loading on that skeletal segment using “hip protectors.” The use of these orthosis has yielded mixed benefits so, for the moment, their use is recommended only in institutionalised patients with a very high risk of falling.

Assessment of the home environment is important. Many obstacles or hazard, like poor lighting, wires or carpets, inadequate footwear, and the presence of pets, are modifiable.

### Integrated approaches for secondary prevention of fractures

The secondary prevention of fragility fractures, aimed at preventing re-fracture, is very complex and all the strategies adopted over the years have yielded disappointing results. In fact, OSMED data, recently published in Italy by AIFA, indicate that approximately 80% of patients with fragility fractures (femoral or vertebral), or chronic treatment with glucocorticoids, do not receive either a correct diagnosis or adequate medical treatment, and that after 1 year, only about 50% of patients continue to follow their therapy correctly. It is, therefore, necessary to develop new integrated and multidisciplinary models, such as Orthopaedic and Geriatric Co-Management, Fracture Unit and Fracture Liaison Services. These are flexible models based on improved communication between the various specialists and the general practitioners involved in the management of patients with fragility fractures. The strength of these multidisciplinary models is their ability to be implemented in the context of very different clinical and organisational systems. The role of nurses with specific expertise in the field of osteoporosis and fragility fractures (Nurse Case Manager or Bone Care Nurses) is essential for them to function properly. It is the nurses who must not only ensure the care of patients with fragility fracture during hospitalisation by fostering proper communications between the orthopaedic team, the various specialists involved and the general practitioner, but from admission on devise an educational programme for patients and their caregivers to ensure proper use of drugs, to improve adherence to treatment and prevent falls [65–70].

## Drug intervention

### Pharmacological thresholds

The treatment of osteoporosis should aim at reducing the risk of fracture in high-risk subjects and the values of the DXA *T*-score, availed of by the WHO to establish diagnostic thresholds, cannot be used to identify pharmacological intervention thresholds. In fact, the risk of fracture should always be obtained by integrating densitometric data with other important clinical factors such as age, steroid therapy, smoking, thinness, etc., combined to determine fracture risk, regardless of BMD. This can be quickly obtained using mathematical algorithms that quantify the risk in terms of “10-year fracture risk” such as FRAX^®^ or DeFRA and which are particularly important if the patient is not fractured.

The history of previous osteoporotic fractures, adjuvant hormonal block in men with prostate cancer and in women with breast cancer, and chronic glucocorticoid therapy, in particular prednisone doses equivalent to ≥ 5 mg/day, are associated with such a high risk of fracture that the decision to initiate drug therapy may rule out the need to acquire densitometric values.

### Anti-osteoporotic drugs

The drugs available in Italy for the treatment of osteoporosis can be divided into two categories: anti-resorptive (or anti-catabolic) and anabolic. All the drugs belonging to these two categories are able to significantly reduce the risk of vertebral fractures, while their ability to reduce risks of non-vertebral and femoral fractures has been demonstrated in only a few cases; their reimbursement by the National Health Service (NHS) is governed by Note 79, and for some of these drugs (denosumab, strontium ranelate, and teriparatide), it is necessary that an authorized specialist Treatment Plan be endorsed. In any case, it is necessary for the physician to aim at ensuring adequate therapeutic adherence by means of appropriate information to patients and the careful choice of the medication prescribed.

#### Anti-catabolic drugs

##### Bisphosphonates

Bisphosphonates (BP) are synthetic analogues of pyrophosphate compounds able to fixate selectively on bony surfaces subject to remodelling. They block osteoclast activity at these locations, reduce bone turnover, and increase bone density with a different mechanism of action as a function of the presence or absence of an amino group. BP is absorbed by the 0.5–5% of the gastrointestinal tract, and is contraindicated in patients with hypocalcaemia, gastrointestinal diseases, renal failure (CCr < 30 mL/min), or if pregnant or nursing.

Etidronate and clodronate are BP lacking amino groups that increase vertebral density and maintain femoral neck density in postmenopausal women.

Etidronate is not indicated in osteoporotic patients, and clodronate was is effective in reducing clinical fractures at a dose of 800 mg/day orally. The anti-fracture efficacy of intramuscular clodronate therapy at the most commonly used dosage in Italy (100 mg/week or 200 mg every 2 weeks) has not been definitively demonstrated, and therefore, it must be regarded as a second-choice drug for the treatment of osteoporosis.

The efficacy of alendronate and risedronate for the prevention of vertebral and non-vertebral fractures (including hip) is extensively documented. Their anti-fracture efficacy has been demonstrated with the daily administration of the two drugs, and it can be used in weekly administrations (70 mg/week of alendronate and 35 mg/week or 75 mg 2 days/month for risedronate) on the basis of the equivalence of different formulations in determining increases in BMD. Recently, in Italy, formulations of alendronate in a liquid form have become available.

Ibandronate is registered based on studies using a dosage of 2.5 mg/day. At this dose, it has proven only effective in reducing the risk of vertebral fractures, and has been subsequently marketed at a dosage of 150 mg/month or 3 mg iv/3 months, or cumulative-bioavailable double doses to those used in the pivotal studies.

Zoledronic acid (5 mg/iv/year) is registered for the treatment of osteoporosis based on a study that documents clearly reduced risk of vertebral, non-vertebral, and hip fractures after 3 years of treatment. In one ancillary study, also a reduction in overall mortality is demonstrated.

Alendronate, risedronate, and zoledronate also have been registered for the treatment of male and corticosteroid-induced osteoporosis.

Neridronate is the only BP indicated for the treatment of *osteogenesis imperfecta,* and in Italy, it is currently indicated for the treatment of algodystrophy (complex regional pain syndrome type I) on the basis of data obtained in a randomised controlled trial.

As for adverse events due to BP, these can be classified as follows:

- Acute Phase Reaction: The administration of amino-BP by the iv route (but also of oral BP in high doses) may be associated with an influenza-like syndrome with a duration of 1–3 days, and characterized by fever and widespread musculoskeletal pain, more frequent, and severe after the first administration of the drug. Its symptoms are well controlled with oral acetaminophen, and only rarely, is it necessary to administer corticosteroids.
- Atypical femoral fractures (AFF): these are transverse stress fractures whose diagnosis requires compliance with precise classification criteria. The incidence of these fractures is very low (3.2–50 cases per 100,000 person/year), but is positively correlated with the duration of treatment with BP. Based on the available data, and given the rarity of these events, the risk/benefit ratio in the use of BP for the prevention of fragility fractures is clearly in favour of the benefit. To minimize the risk of AFF in patients treated with BP, it may be useful to: (a) consider periods of “therapeutic vacation,” after careful assessment of the benefit–risk ratio; (b) monitor and correct other risk factors for atypical fractures (chronic use of glucocorticoids, hypovitaminosis D, chronic use of proton pump inhibitors, and presence of skeletal diseases other than osteoporosis).
- ONJ (OsteoNecrosis of the Jaw) or osteomyelitis of the jaw. This event is very rare in patients who use BP for the treatment of osteoporosis (1:10,000 patients treated), but it increases if they are subjected to oral cavity interventions with bone tissue exposure. In patients starting treatment with BP for osteoporosis, there is no need for prior dental examination and treatment. In cases of invasive dental surgery (extraction), we recommend the use of topical antiseptics (chlorhexidine mouthwash 0.20%) and antibiotics (amoxicillin, optionally in combination with metronidazole) 2 days prior to surgery, and for 6–8 days after, especially if there are individual risk factors (diabetes, immunosuppression, use of steroids, smoking, and alcohol), while a brief suspension of the BP is carried out.

##### Duration of bisphosphonate therapy

In view of the adverse events associated with long-term therapy with BP, the need for continued treatment should be reviewed at regular intervals. Based on available data, risk reassessment should be carried out after 5 years of treatment with alendronate, ibandronate, and risedronate and after 3 years for treatment with zoledronate. Suspending treatment for 12–24 months in patients who have received oral BP for over 5 years and are at low risk of fracture is advisable. However, continuation of treatment up to 10 years (maximum duration of treatment hitherto investigated) in patients at high risk of fracture, such as those with femoral *T*-score < − 2.5 or with prior vertebral fractures and *T*-score femur less than − 2.0 is recommended. In high-risk patients treated with zoledronate, continued treatment with zoledronate for other 3 years is indicated.

##### Denosumab

Denosumab is a human monoclonal antibody capable of neutralising RANKL, a cytokine that interacts with the RANK receptor on the membrane of preosteoclasts and mature osteoclasts, affecting their recruitment, maturation, and survival. Pivotal studies were conducted using 60 mg of subcutaneous denosumab every 6 months. This dose ensures almost total suppression of bone turnover, and determines an increase in BMD higher than that obtainable with BP both in trabecular and cortical bone with a consequent reduction of fragility fractures at all skeletal sites. Denosumab is effective in reducing the risk of fractures in women with breast cancer treated with aromatase inhibitors, as well as in men with prostate cancer being treated with anti-androgens. In very severe cases of established osteoporosis, the combination denosumab/teriparatide therapy has resulted in a marked increase in BMD. Similar advantages in terms of increase in BMD are obtained with sequential teriparatide–denosumab therapy. Unlike BP, discontinuation of treatment with denosumab is followed by an abrupt increase of bone turnover, and by a rapid loss of BMD. Therefore, the suspension of denosumab generally requires the patient to begin, as soon as possible, the treatment with BP at an adequate dosage.

Treatment with denosumab may, sometimes, cause hypocalcaemia; therefore, this must be corrected and prevented by adequate intake of calcium and vitamin D. In the postregistration extension studies, rare cases of ONJ and AFF were seen.

##### Hormone replacement therapy (HRT)

Menopausal women undergoing oestrogen treatment, on its own or in combination with progestin, and with tibolone, are able to reduce bone turnover and increase bone mass. Oestrogen anti-fracture efficacy has been confirmed by several randomised trials and major observational studies (especially the WHI study). Despite the positive effect on fractures, to which may be added a reduction in the risk of colorectal cancer, these drugs entail an increased risk of breast cancer, stroke and thromboembolic events. Therefore, HRT is no longer indicated for the treatment or prevention of osteoporosis. For women suffering from the climacteric syndrome, especially if still within the 50–55 age-range, temporary administration of oestrogen or oestrogen plus progestin (depending on whether or not there is an intact uterus) may be considered in some way to be physiological and be proposed, it is also effective for the prevention of osteoporosis.

##### Selective oestrogen receptor modulators (SERMs)

SERMs are synthetic compounds capable of binding with oestrogen receptors that produce agonist effects at bone and liver level, and antagonist effects at breast and genitourinary tract level. The SERMs currently approved in Italy for the prevention and treatment of osteoporosis are raloxifene and bazedoxifene. The pivotal trial MORE raloxifene (60 mg/day) reduced the incidence of new vertebral fractures, (but not those of non-vertebral and femoral fractures) and invasive breast cancer, accentuating vasomotor phenomena in some patients.

Bazedoxifene (20 mg/day) significantly reduces the risk of vertebral and non-vertebral fractures (but not hip fractures) in high risk of fracture women treated for 3–5 years. Compared to raloxifene, bazedoxifene has a higher anti-oestrogenic effect in the uterus in the absence of significant side effects. SERMs, like HRT, are associated with increased risk of thromboembolic events, and are not recommended in patients who have had, or who are at risk of venous thrombosis [23, 71–91].

#### Anabolic drugs

##### Teriparatide

The administration of parathyroid hormone, and in particular, of its active fragment 1–34 (teriparatide), stimulates both bone formation and resorption, with a predominant effect on neoformation (anabolic window) that is evident especially during the first 12 months of treatment. Increases observed in BMD values are significantly higher than those obtained with BP in trabecular bone only, with an increase of close to 10%, in spinal BMD at 18 months. However, treatment with teriparatide also determines an improvement in some geometrical characteristics of cortical bone related to resistance to fracture.

Teriparatide (20 µg/day sc) has proved capable of reducing vertebral and non-vertebral fractures in postmenopausal women, and currently, its administration cannot exceed a total of 24 months. On withdrawal of treatment, there is a rapid decline in densitometric values; therefore, it is advisable to start anti-resorptive therapy as soon as possible. Due to its high cost, it is reimbursed by the NHS for secondary prevention in patients with osteoporosis at high risk of fracture or “non-responsive” to anti-resorptive medications. Treatment with teriparatide is frequently associated with less severe disorders (nausea and cramps in the lower limbs) and increased incidence of hypercalcemia, usually asymptomatic. Teriparatide is contraindicated in patients with hyperparathyroidism, Paget’s disease of bone, severe renal failure, primary tumours, or skeletal metastasis or previous radiation therapy on the skeleton.

#### Dual-action drugs

##### Strontium ranelate

Treatment with strontium ranelate is effective when seeking to reduce risks of vertebral, non-vertebral, and hip fractures in postmenopausal women with osteoporosis. This drug increases bone formation markers, and modestly decreases those for resorption. Increases in densitometry registered during therapy are approximately 50% related to the higher molecular weight of strontium as compared to calcium. Since treatment with strontium ranelate has also been associated with an increased risk of myocardial infarction and thromboembolic events, it is contraindicated in patients with ischaemic heart disease, peripheral arterial disease, or cerebrovascular disease, or a history of, or uncontrolled high blood pressure. Rare cases have been reported of serious allergic skin reactions, sometimes associated with potentially fatal systemic symptoms such as DRESS (Drug Rash with Eosinophilia and Systemic Symptoms) and toxic epidermal necrolysis. The use of strontium ranelate is currently restricted to the treatment of severe osteoporosis in postmenopausal women or adult men at high risk of fractures, for whom treatment with other medicines approved for osteoporosis therapy is not feasible [92–94]

## Kyphoplasty and vertebroplasty

Vertebral fractures often occur with sudden and rapidly progressing pain, not in relation to effective trauma, initially continuous, also felt at rest, and then when load-bearing.

The treatment of vertebral fractures in the acute stage involves conservative measures such as rest, use of corsets, and minor and major painkillers. Pain due to a vertebral fracture usually begins to fade after 1–3 weeks, and disappears completely after a few months. In some cases, however, the pain can last for months in relation to the severity and location of the fractured vertebra, which influences the development or persistence of biomechanical instability.

Transpedicular injections of a synthetic material similar to cement within the fractured vertebral body may be accompanied by immediate cessation of pain.

The methods currently proposed to stabilize or reduce–stabilize vertebral fractures are vertebroplasty, in which cement under high pressure is injected with greater risk of leakage and pulmonary embolism, and kyphoplasty, in which the cement is introduced at low pressure with lower risk of leakage after the introduction of a balloon which is then inflated within the vertebral body often enabling a partial reduction of the deformity.

Vertebroplasty or kyphoplasty can only be recommended for patients with intractable pain for weeks, with due consideration of the potential risks associated with the procedures and the uncertain benefits in the long term. The use of these procedures is, therefore, not indicated in patients with few or no symptoms.

However, it is essential that all patients with vertebral fragility fractures treated with vertebroplasty or kyphoplasty are prescribed a suitable pharmacological treatment, so that the presence of cement within the vertebral body, when there are systemic conditions for bone fragility, does not expose adjacent vertebrae to an increased risk of fracture [23, 95, 96].
